# Lipocalin-2 in preoperative cerebrospinal fluid is a biomarker for postoperative delirium after hip fracture surgery in older adults: a prospective cohort study

**DOI:** 10.3389/fneur.2025.1653407

**Published:** 2025-09-12

**Authors:** Ning Kang, Xiaoguang Han, Taotao Liu, Jie Huang, Zhuzhu Li, Zhengqian Li, Yi Yuan, Yanan Song, Ning Yang, Xiangyang Guo

**Affiliations:** ^1^Department of Anesthesiology, Peking University Third Hospital, Beijing, China; ^2^Department of Spine Surgery, Beijing Jishuitan Hospital, Beijing, China; ^3^Department of Spine Surgery, Peking University Fourth School of Clinical Medicine, Beijing, China; ^4^Beijing Key Laboratory of Robotic Orthopaedics, Beijing, China; ^5^Department of Orthopedics, Peking University Third Hospital, Beijing, China; ^6^Department of Anesthesiology, Beijing Jishuitan Hospital, Beijing, China; ^7^Beijing Center of Quality Control and Improvement on Clinical Anesthesia, Beijing, China

**Keywords:** hip fracture, elderly patient, lipocalin-2, postoperative delirium, interleukin-6

## Abstract

**Background:**

Postoperative delirium (POD) is a common central nervous system complication in older adult surgical patients. At present, the mechanism for POD is still unclear. Lipocalin-2 (LCN2) may have an impact on cognitive function, but the relationship between LCN2 and POD has remained unclear. Therefore, we sought to investigate the relationship between the levels of LCN2 in plasma and cerebrospinal fluid (CSF) and the occurrence of POD in older adults undergoing hip fracture surgery.

**Methods:**

We conducted a prospective observational cohort study involving 186 older adults (≥65 years old) who underwent hip fracture surgery under spinal anesthesia. CSF and blood samples were collected. The levels of LCN2, interleukin-6 (IL-6), and interleukin-1 (IL-1) were measured using an enzyme-linked immunosorbent assay (ELISA). We used the 3-min diagnostic interview to evaluate delirium defined by the Confusion Assessment Method (3D-CAM), to screen for POD, and the Memorial Delirium Assessment Scale (MDAS) to evaluate the severity of delirium. Multivariable logistic regression was applied to identify independent predictive factors for POD. The relationship between CSF LCN2 levels and POD risk was assessed through receiver operating characteristic (ROC) curve analysis. Correlation analysis was used to investigate the association between CSF LCN2 and MDAS scores as well as IL-6.

**Results:**

Among the 186 patients ultimately included, 29 (15.6%) developed POD. Their preoperative CSF LCN2 level was significantly higher than that of those without POD (*p* = 0.001). The multivariable logistic regression analysis revealed that an elevated preoperative CSF LCN2 level [odds ratio (OR) 2.546, 95% confidence interval (CI) 1.345–4.822; *p* = 0.004] was an independent predictor of POD. Moreover, among POD group patients, preoperative CSF LCN2 levels were positively correlated with the MDAS scores (*r* = 0.688, *p* < 0.001) and CSF IL-6 levels (*r* = 0.379, *p* = 0.043). ROC analysis of preoperative CSF LCN2 showed an area under the curve of 0.713 (95% CI 0.615–0.810) with a specificity of 75.0%, and sensitivity of 58.6%.

**Conclusion:**

Elevated preoperative CSF LCN2 levels are associated with an increased risk and severity of POD in older adults undergoing hip fracture surgery.

**Clinical trial registration:**

https://www.chictr.org.cn/, ChiCTR2200061407.

## 1 Introduction

Hip fracture is a major disease threatening the health and safety of adults aged 65 years or older. The incidence increases with the aging trend of the population (1). Fracture reduction and internal fixation surgery are the main treatment methods for hip fractures. Postoperative delirium (POD) is a common complication in such patients (2). POD is an acute fluctuating mental state change that occurs in patients after surgery under anesthesia, often accompanied by decreased consciousness, attention disorders, psychomotor disorders, and sleep–wake cycle disorders. According to the patient’s mental activity level, it can be categorized into indifferent, restless, and mixed types (3). POD often occurs within 7 days after surgery or before discharge, and the symptoms can last from hours to weeks (4). The incidence is approximately 4–61% (5), which can increase mortality, hospital costs, and hospital stay (6), constituting a major public health problem.

At present, the mechanism for delirium has remained unclear and is the result of a combination of multiple factors (7, 8), including the neuroinflammatory hypothesis. Central nervous system (CNS) inflammation is closely related to the occurrence of POD (9).

Elevated levels of IL-6 in preoperative CSF are associated with the occurrence of postoperative delirium (10, 11). Another key molecule implicated in neuroinflammation is LCN2, also known as neutrophil gelatinase-associated lipocalin (NGAL), belonging to the lipocalin family (12). Although NGAL is expressed in various peripheral tissues (13–19), its role within the CNS has garnered substantial attention. Under physiological conditions, LCN2 expression in the brain is low (20). However, in response to neuroinflammation, its expression is markedly upregulated (21, 22), primarily in activated astrocytes (23), neuron (24, 25), microglia (24), and endothelial cells (26). Mosialou et al. reported that bone-derived LCN2 can cross the blood–brain barrier (BBB) and bind to melanocortin-4 receptors on hypothalamic neurons (27). However, current research on the mechanism of its crossing the BBB is limited. Two primary mechanisms have been proposed, though neither is fully elucidated. First, it may be facilitated by inflammatory conditions that increase BBB permeability (28, 29). Second, the possibility of receptor-mediated transport exists (29). Within the CNS, LCN2 exerts pleiotropic effects (30). It is implicated in iron homeostasis (31), synaptic plasticity (32), and neuronal apoptosis (33). Crucially, LCN2 acts as a key mediator in the neuroinflammatory cascade, often acting with downstream pyroptosis to promote proinflammatory responses (34). Its involvement in the pathophysiology of various neurodegenerative disorders, including Alzheimer disease and Parkinson disease, is well-documented (35–37). Notably, alterations in LCN2 concentrations in CSF have been linked to cognitive impairment (38, 39), suggesting its potential as both a biomarker and a therapeutic target for neurological conditions.

LCN2 may be involved in the occurrence of postoperative cognitive dysfunction (40, 41), and a correlation between LCN2 in plasma and POD is suggested (42). However, clinical studies are limited, and to our knowledge, the correlation between LCN2 and POD in CSF has not been examined. Therefore, we sought to determine the relationship between LCN2 levels in plasma and CSF and the occurrence of POD.

## 2 Materials and methods

### 2.1 Research design and ethical approval

This study was a single-center, prospective observational cohort study conducted at Jishuitan Hospital in Beijing. The protocols were rigorously reviewed and approved by the Ethics Committee of Beijing Jishuitan Hospital (Institutional Review Committee: JLKS202204-08; International Clinical Website Registration Number: ChiCTR2200061407). Research protocols strictly adhered to the principles outlined in the Declaration of Helsinki and its latest amendment (2013). All participants or their legally authorized representatives documented their written informed consent by signing a form before enrollment and undergoing any research-related procedures. This study followed the STROBE guidelines for reporting observational epidemiological studies (43).

### 2.2 Patients and setting

**Figure 1:**
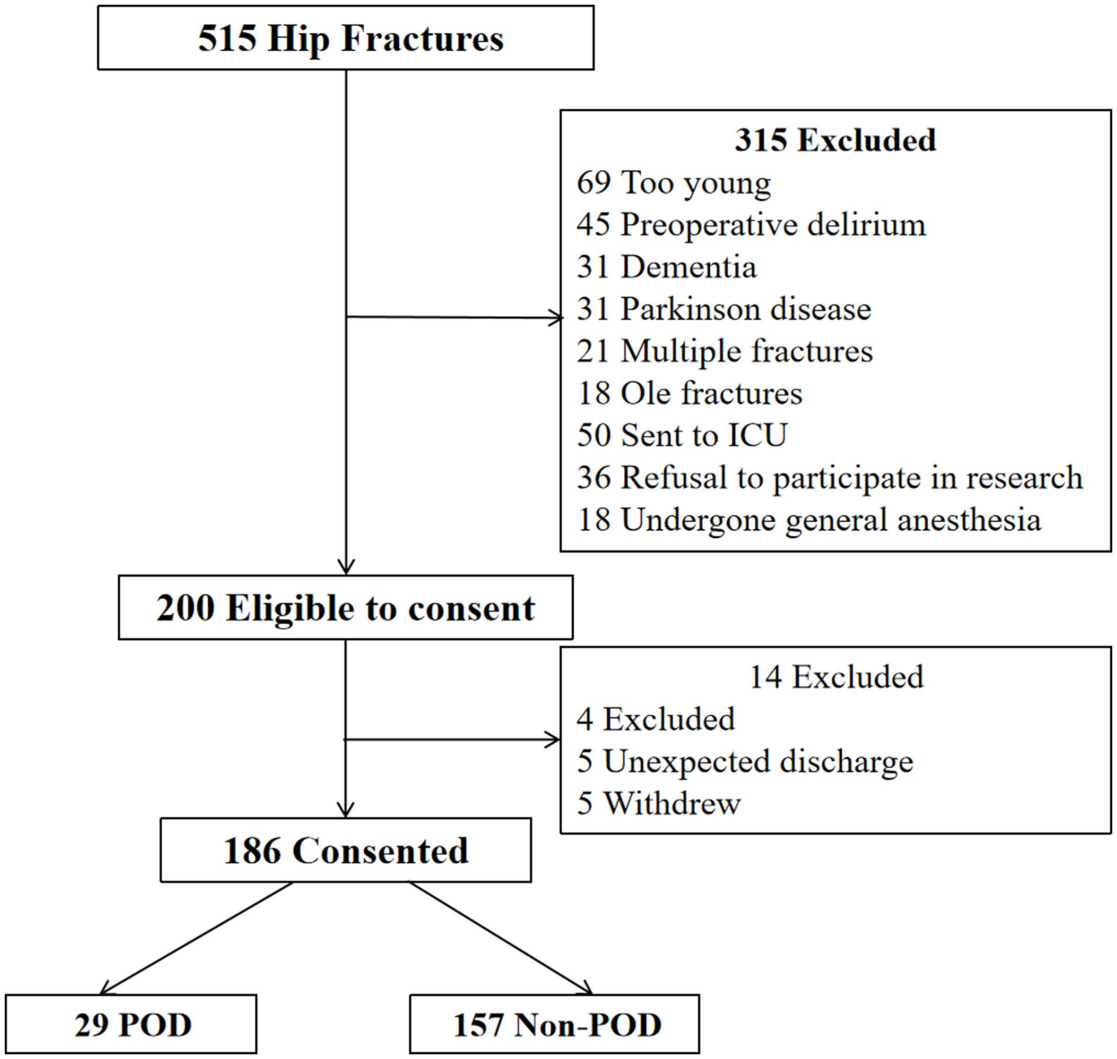
Flow diagram. We initially screened 515 patients for the study, and 186 patients were ultimately included in the data analysis. MMSE, Mini-Mental State Examination; ICU, intensive care unit; POD, postoperative delirium.

We recruited patients aged 65 years and over who planned hip fracture surgery under spinal anesthesia between March 2023 and December 2023. All patients were admitted to the orthopedic ward for older adult patients. Inclusion criteria: diagnosed hip fracture site is only unilateral; age ≥65 years; American Society of Anesthesiologists (ASA) Physical Status Classification System levels I, II, and III; no history of allergy to anesthetic drugs; agreed to be a patient participant. Exclusion criteria: those who have not undergone surgery within 48 h after admission; severe comorbidities in other systems; contraindications for nerve block (needle site infection, local anesthesia drug allergy, coagulation dysfunction, international standardized ratio>1.4, platelet count <80 × 10^9^); pathological fracture; metabolic bone disease; old fractures; history of stroke within 6 months before surgery; dementia, mental illness, and preoperative delirium; alcohol dependence or drug addiction; transferred to an intensive care unit (ICU) after surgery, with aphasia and hearing impairment; patients with Parkinson disease. We initially screened 515 patients, and ultimately included 186 in the present study. A flow diagram of the patient recruitment process is shown in Figure 1.

### 2.3 Data collection

We visited the patients 1 day before the surgery and collected baseline data, including age, sex, body mass index (BMI), ASA grading, Mini-Mental State Examination (MMSE) score, Age-adjusted Charlson Comorbidity Index (ACCI) score, Pittsburgh Sleep Quality Index (PSQI) score, and smoking status. Based on the patient’s medical records, other information was also collected, including comorbidities, medication use, medical history, fracture classification, anesthesia and surgical type, and time from injury to surgery. All medical history collection, physical assessment, and cognitive assessment related to dementia were conducted by physicians specializing in geriatrics.

### 2.4 Anesthesia and analgesia

As a key component of our standardized protocol designed to minimize confounding variables, all enrolled patients underwent spinal anesthesia as their sole method of anesthesia. This choice was based on institutional preference and clinical evidence suggesting potential benefits for older patients with hip fracture, including effective pain control and a possibly reduced incidence of POD compared with that with general anesthesia (44–46). After the patients entered the operating room, electrocardiogram monitoring, pulse oximetry, and invasive blood pressure monitoring via radial artery catheterization were initiated. Before positioning, the high iliac fascial space tissue (“funnel sign”) was identified on the affected side, and each patient was given 30 mL of 0.33% ropivacaine. Subsequently, the patient was placed in a lateral position with the affected side facing upwards, and a dose of 8–10 mg of 0.3% ropivacaine was administered at the L3–L4 intervertebral space. The anesthesia level reached T10, and no intravenous sedatives or anticholinergic drugs were used during the operation. Standard aseptic techniques were employed. Postoperative patient-controlled analgesia (PCA) was administered intravenously, with a specific medication regimen of 100 μg of sufentanil, 200 mg of flurbiprofen axetil, 10 mg of tropisetron hydrochloride, and 100 mL of physiological saline (background infusion rate of 2 mL/h, PCA compression dose of 0.5 mL, locking time of 15 min). For patients in generally poor condition, the dosage of medications was adjusted as appropriate. The analgesic pump was removed after the dosage was fully delivered. For patients who required additional analgesics, intramuscular injection of duromeprazole (50 mg) or oral administration of acetaminophen could be given according to the patient’s condition to alleviate pain.

### 2.5 Delirium assessment

We used the 3-min diagnostic interview to evaluate delirium defined by the Confusion 3D-CAM (47), to screen for POD, and the MDAS (48) to evaluate the severity of delirium. This evaluation can be completed within an average of 3 min and has excellent performance compared with other methods of evaluation. The evaluation mainly includes the following four characteristics: acute onset and fluctuating condition; inability to concentrate; disordered thinking; and change in consciousness level. Specialists in geriatrics trained by professional psychiatrists administered the 3D-CAM to patients twice daily (in the morning and afternoon) during the first two postoperative days.

### 2.6 Blood sample and CSF collection

We collected 4 mL arterial blood samples from all patients before inducing anesthesia and immediately after surgery. Blood samples were drawn into EDTAK_2_-containing tubes (BD Biosciences, San Jose, CA, United States) kept at 4°C and centrifuged (3,000 × *g* for 10 min) within 4 h to separate plasma and blood cells. All plasma samples were stored at −80°C and sent to the laboratory of Peking University Third Hospital for further processing.

Before administering local anesthesia for a subarachnoid block, we slowly extracted 2 mL of CSF using a syringe and placed it in a sterile polypropylene tube. The CSF was immediately centrifuged (3,000 × *g* for 10 min) at 4°C to remove cells (49). Then we separated and reserved the supernatant, which was stored at −80°C until assayed.

### 2.7 Biochemical analysis

We used an enzyme immunoassay (Proteintech, Chicago, IL, United States) to measure the concentration of LCN2, with a detection limit of 0.0781 ng/mL. The levels of indicators such as albumin, creatinine, aspartate aminotransferase (AST), and alanine aminotransferase (ALT) were measured using a blood biochemistry analyzer (Hitachi, Tokyo, Japan); white blood cell (WBC), red blood cell (RBC), platelet, and hemoglobin levels were measured using a blood analyzer (Sysmex, Kobe, Japan). IL-1 *β* and IL-6 levels were also measured using enzyme immunoassays (Boster, Wuhan, China).

### 2.8 Participant sample size

In the present study, a binary logistic regression model was used to analyze the correlation between LCN2 content in preoperative CSF and POD. Five events per variable (EPV) is the widely used minimum standard for sample size analysis (50). It was estimated that five variables could be included in the final model. For a given number of EPVs, 5 EPV events were required to analyze the sample. At least 25 events (patients with POD) were necessary for analysis. Factoring in a dropout rate of 10%, 28 POD patients were needed. Moreover, a previous study showed that approximately 16% of patients undergoing hip replacement surgery developed POD (46), hence, the minimum sample size was 175.

### 2.9 Statistical analysis

All statistical analyses were performed using IBM SPSS Software for Windows (version 25.0; IBM Corp., Armonk, NY) and GraphPad Prism (version 8.0; GraphPad Software, San Diego, CA). A Shapiro–Wilk test was used to assess the normality of continuous data distributions. Normally distributed continuous data are presented as mean ± standard deviation (SD) and were compared using an independent samples Student *t*-test. Non-normally distributed data are reported as median and interquartile range (IQR) and were compared using a Mann–Whitney *U*-test. Categorical data are expressed as frequency (*n*) and percentage (%) and were compared using chi-square or Fisher exact tests, as appropriate. To identify independent predictors for POD, variables with clinical relevance or a *p* < 0.10 in univariable analysis were entered into a multivariable binary logistic regression model using a forward conditional method. Odds ratios (ORs) and corresponding 95% confidence intervals (CIs) were calculated. Given a non-normal distribution of the data, a Spearman rank correlation coefficient was used to assess the association between preoperative CSF LCN2 levels and other variables (CSF IL-6, MDAS score). The predictive performance of preoperative CSF LCN2 for POD was evaluated by constructing a receiver operating characteristic (ROC) curve. The area under the curve (AUC) and its 95% CI were calculated. The Youden index (J = sensitivity + specificity − 1) was used to determine the optimal cutoff value. For all analyses, a two-sided *p* < 0.05 was considered significant.

## 3 Results

### 3.1 Participant characteristics

We screened 515 patients with hip fractures for the present study. As shown in the patient flowchart (Figure 1), 315 patients were excluded, mainly owing to their age being under 65 years (*n* = 69), pre-existing delirium (*n* = 45), and diagnosis of dementia (*n* = 31). Therefore, 200 patients met the criteria and agreed to participate. After further exclusion owing to accidental discharge or withdrawal of consent, a cohort of 186 patients was ultimately included and completed the study protocol. Among these 186 patients, 29 (15.6%) developed POD after surgery, while the remaining 157 patients did not, forming the non-POD group.

The patients in the POD group were significantly older than those in the non-POD group (median age: 80.0 years vs. 76.0 years, *p* = 0.018). Before surgery, those in the POD group showed significantly poorer cognitive function, with lower MMSE scores (median: 25.0 vs. 26.0, *p* = 0.005) and higher ACCI scores (mean: 5.14 vs. 4.41, *p* = 0.005). In addition, the prevalence of diabetes in the POD group was significantly higher (48.28% vs. 28.66%, *p* = 0.037), and the proportion of insulin or other hypoglycemic drugs was also higher (44.83% vs. 24.84%, *p* = 0.028). We found no significant differences between the two groups in terms of sex, BMI, ASA classification, Activities of Daily Living (ADL) score, PSQI score, or preoperative pain score (Table 1).

**Table 1:** Patients’ baseline characteristics and intraoperative and postoperative data.

Variable	Non-POD group (*n* = 157)	POD group (*n* = 29)	*p*
Age, years	76.0 (70.0,85.0)	80.0 (76.5,86.0)	0.018*
Sex, female, n (%)	119 (75.8)	23 (79.3)	0.682
BMI, kg/m^2^	23.2 ± 3.9	23.1 ± 3.5	0.827
ASA physical status class			
II, n (%)	69 (59.5)	18 (62.1)	0.533
III, n (%)	47 (40.5)	11 (37.9)	
MMSE score	26.0 (25.0,26.0)	25.000 (24.0,26.0)	0.005**
ADL score	100.0 (90.0,100.0)	100.0 (80.0,100.0)	0.237
PSQI score	14.0 (10.0,20.0)	16.0 (13.5,20.0)	0.070
ACCI score	4.4 ± 1.3	5.1 ± 1.3	0.005**
Resting NRS	2.4 ± 1.1	2.5 ± 1.2	0.701
Motion NRS	5.0 (4.0,6.5)	5.0 (5.0,6.0)	0.460
Smoke, n (%)	26 (16.6)	2 (6.9)	0.181
Hypertension, n (%)	98 (62.4)	19 (65.5)	0.751
Diabete, n (%)	45 (28.7)	14 (48.3)	0.037*
Ischemic heart disease, n (%)	30 (19.1)	5 (17.2)	0.813
Chronic obstructive pulmonary disease, n (%)	15 (9.6)	2 (6.9)	0.648
Stroke, n (%)	30 (19.1)	5 (17.2)	0.813
Using β-blockers, n (%)	18 (11.5)	4 (13.8)	0.721
Using calcium channel blocker, n (%)	69 (44.0)	12 (41.4)	0.798
Using ACEI/ARB, n (%)	38 (24.2)	5 (17.2)	0.414
Using statins, n (%)	44 (28.0)	9 (31.0)	0.742
Using insulin/hypoglycemic drugs, n (%)	39 (24.8)	13 (44.8)	0.028*
Preoperative laboratory examination			
WBC, ×10^9^/L	9.7 (7.6, 11.6)	9.5 (8.4, 10.9)	0.963
RBC, ×10^12^/L	3.9 ± 0.6	4.0 ± 0.6	0.481
PLT, ×10^9^/L	194.0 (146.0, 236.0)	190.0 (158.5, 243.0)	0.774
Glycosylated hemoglobin, %	6.0 (5.6, 6.5)	6.1 (5.8, 6.7)	0.387
tP1NP, μg/L	41.0 (31.6, 56.0)	47.3 (31.4, 71.7)	0.160
β-CTX, pg./mL	0.3 (0.2, 0.5)	0.4 (0.2, 0.5)	0.902
25-OHVD, ng/mL	17.2 (12.2, 23.8)	14.8 (11.4, 21.8)	0.121
Albumin, g/L	41.3 (38.6, 43.7)	41.2 (39.3, 43.0)	0.707
Creatinine, mmol/L	53.0 (44.0, 66.0)	56.0 (46.5, 74.0)	0.238
ALT, U/L	15.0 (12.0, 19.0)	14.0 (11.0, 20.5)	0.964
AST, U/L	18.0 (15.5, 23.0)	17.0 (15.5, 23.0)	0.768
PaO2, mmHg	79.0 (70.0, 88.0)	75.0 (68.0, 86.5)	0.391
Lactic acid, mmol/L	1.0 (0.8, 1.6)	1.2 (0.9, 2.0)	0.305
Intraoperative data			
Anesthesia time, minutes	75.0 (72.5, 90.0)	75.0 (72.5, 92.5)	0.895
Surgery time, minutes	60.0 (60.0, 70.0)	60.0 (60.0, 65.0)	0.532
Blood loss, mL	200.0 (100.0, 200.0)	200.0 (100.0, 200.0)	0.791
Postoperative laboratory examination			
WBC, ×10^9^/L	10.3 (7.8, 12.3)	11.6 (9.7, 13.7)	0.012*
RBC, ×10^12^/L	3.6 (3.3, 3.9)	3.5 (3.3, 4.1)	0.928
PLT, ×10^9^/L	172.0 (133.0, 231.0)	199.0 (156.0, 223.5)	0.380
Postoperative data			
Subjective sleep rating	6.0 (4.0, 7.0)	7.0 (3.0, 8.0)	0.697
Resting NRS	2.0 (1.0, 3.0)	3.0 (1.0, 3.0)	0.073
Motion NRS	4.0 (4.0, 5.0)	4.0 (3.0, 6.0)	0.746
Remedial analgesia, n (%)	100 (63.7)	23 (79.3)	0.103

The categorical variables are expressed as n (%). Normal data are given as mean ± SD, whereas non-normaldata are expressed as median (25th percentile, 75th percentile). BMI, body mass index; ASA, American Society of Anesthesiologists Classification; MMSE, Mini-Mental State Examination; ADL, Activities of Daily Living; PSQI, Pittsburgh Sleep Quality Index; ACCI, Age-adjusted Charlson Comorbidity Index; NRS, numeric rating scale; ACEI, angiotensin-converting enzyme inhibitors; ARB, angiotensin II receptor blockers; tP1NP, total procollagen type I N-terminal propeptide; β-CTX, β-C-terminal telopeptide of type I collagen; *25-OHVD,* 25-hydroxy vitamin D; WBC, white blood cells; RBC, red blood cells; PLT, platelets; ALT, alanine aminotransferase; AST, aspartate transaminase; PaO_2_, oxygen partial pressure. *p < 0.05, **p < 0.01.

### 3.2 CSF concentrations of LCN2, IL-1, and IL-6

**Figure 2:**
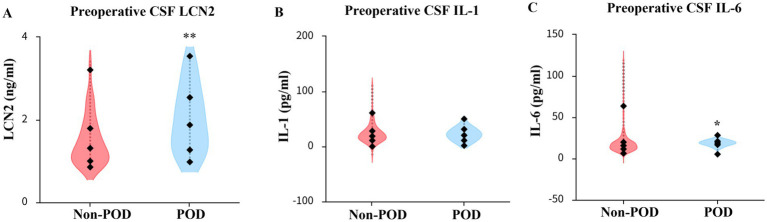
Comparison of LCN2, IL-1, IL-6 concentration in CSF between POD group and non-POD group patients. **(A)** Comparison of reoperative CSF LCN2 concentration between POD group and non-POD group patients. **(B)** Comparison of preoperative CSF IL-1 concentration between POD group and non-POD group patients. **(C)** Comparison of preoperative CSF IL-6 concentration between POD group and non-POD group patients. CSF, cerebrospinal fluid; LCN2, lipocalin-2; IL-1, interleukin-1; IL-6, Interleukin-6; POD, postoperative delirium. *p < 0.05, **p < 0.01.

The median preoperative CSF LCN2 concentration in the POD group was 1.8 ng/mL (IQR 1.3, 2.6), which was significantly higher than the observed 1.3 ng/mL (IQR 1.0, 1.8) in the non-POD group (*p* = 0.001) (Table 2; Figure 2). The preoperative IL-6 concentration in the CSF of patients in the POD group were significantly higher than those in the non-POD group (median: 16.135 vs. 19.248 pg./mL, *p* = 0.031), whereas there was no difference in the preoperative IL-1 concentration between the two group patients (*p* = 0.688) (Table 2; Figure 2).

**Table 2:** Comparison of LCN2 concentration in plasma and CSF between the POD group and non-POD group patients, as well as comparison of IL-1 and IL-6 in CSF between the two groups of patients.

Variable	Non-POD group	POD group	*p*
Preoperative plasma LCN2 (ng/mL)	73.9 (58.2, 98.4)	89.2 (70.5, 106.7)	0.067
Postoperative plasma LCN2 (ng/mL)	110.5 (78.1, 178.9)	102.9 (80.4, 157.6)	0.649
D-value of plasma LCN2 (ng/mL)	22.2 (3.3, 72.1)	6.1 (−2.2, 37.0)	0.084
Preoperative CSF LCN2 (ng/mL)	1.3 (1.0, 1.8)	1.8 (1.3, 2.6)	0.001**
Preoperative CSF IL-1 (pg/mL)	19.9 (12.3, 28.7)	21.7 (11.8, 32.1)	0.688
Preoperative CSF IL-6 (pg/mL)	16.1 (11.9, 20.8)	19.2 (16.7, 21.5)	0.031*

Non-normaldata are expressed as median (25th percentile, 75th percentile). CSF, cerebrospinal cfluid; LCN2, lipocalin-2; IL-1, interleukin-1; IL-6, interleukin-6; POD, postoperative delirium. *p < 0.05, **p < 0.01.

### 3.3 Plasma concentrations of LCN2

**Figure 3:**
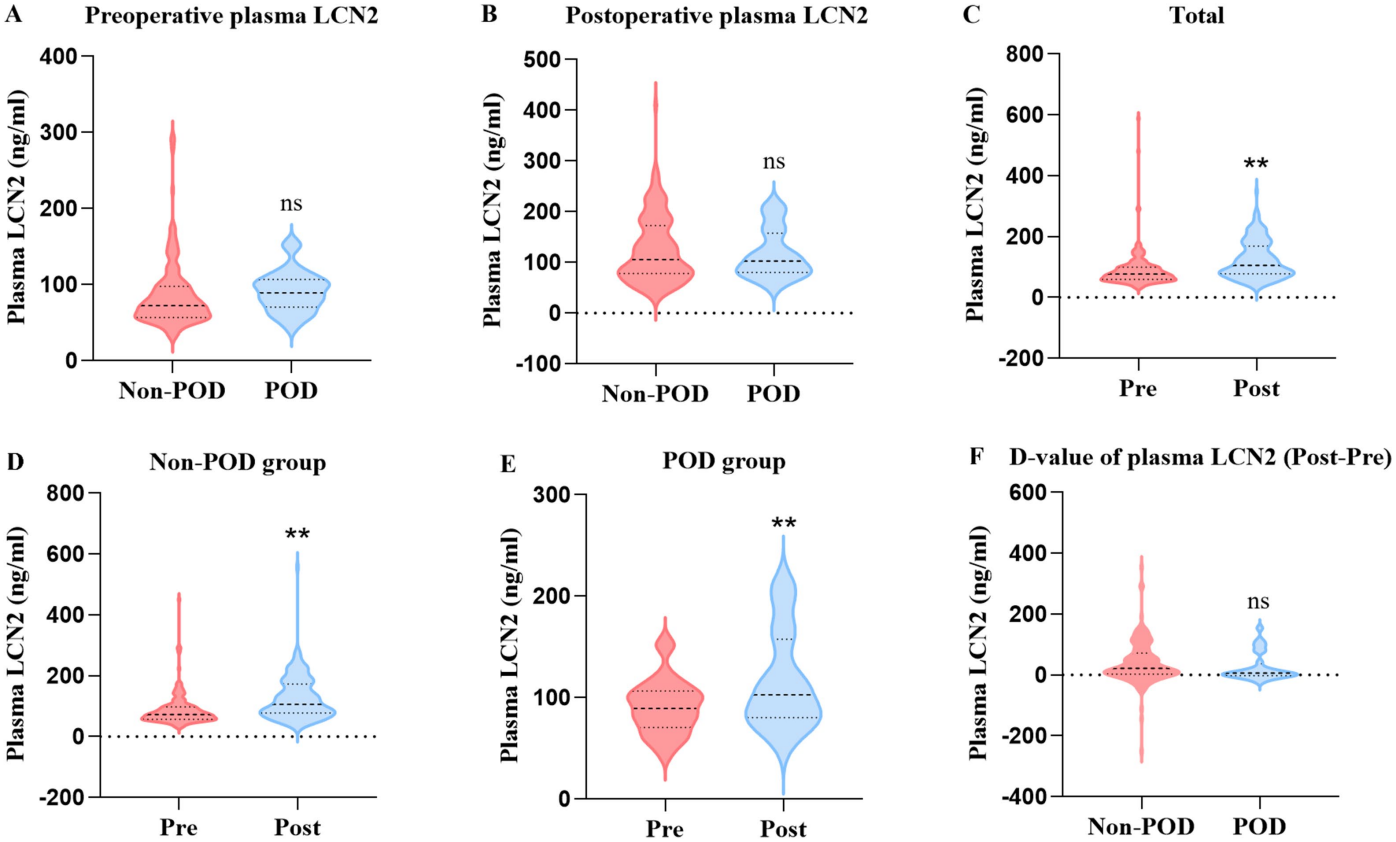
Comparison of preoperative and postoperative plasma LCN2 among total patients, POD group patients, and non-POD group patients. **(A)** Comparison of preoperative plasma LCN2 concentration between POD group and non-POD group patients. **(B)** Comparison of postoperative plasma LCN2 concentration between POD group and non-POD group patients. **(C)** Comparison of LCN2 concentration in postoperative and preoperative plasma in total patients. **(D)** Comparison of LCN2 concentration in postoperative and preoperative plasma in non-POD group patients. **(E)** Comparison of LCN2 concentration in postoperative and preoperative plasma in POD group patients. **(F)** Comparison of D-value of postoperative and preoperative plasma LCN2 concentration between the POD group and non-POD group patients. LCN2, lipocalin-2; POD, postoperative delirium. **p < 0.01.

There was no statistically significant difference in preoperative and postoperative plasma LCN2 concentrations between the POD group and the non-POD group (Table 2; Figure 3). To investigate the systemic effects of surgery on LCN2, we compared the plasma LCN2 levels before and after surgery. There was a highly significant increase in postoperative plasma LCN2 concentration (mean: preoperative 93.72 ng/mL vs. postoperative 132.9 ng/mL, *p* < 0.01) among total patients. A significant increase in postoperative concentration was observed in both the non-POD group (*p* < 0.01) and the POD group (*p* < 0.01). We further compared the D-value in LCN2 concentration in postoperative and preoperative plasma between non-POD and POD groups, and found no difference (Table 2; Figure 3).

### 3.4 Binary logistic regression analysis

**Figure 4:**
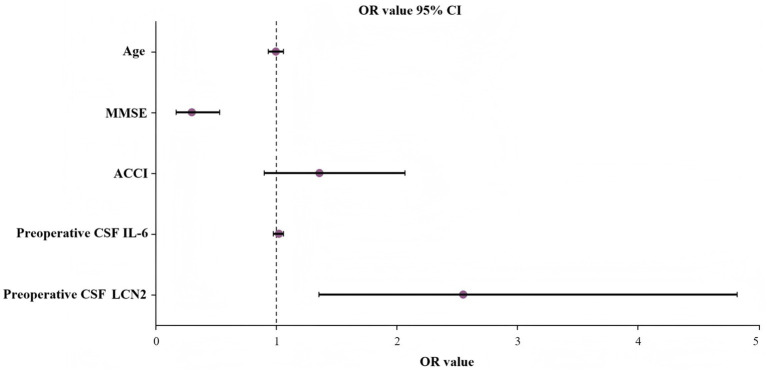
Predictors of POD with OR and 95% CI. MMSE, Mini-Mental State Examination; ACCI, Age-adjusted Charlson Comorbidity Index; CSF, cerebrospinal fluid; IL-6, interleukin-6; POD, postoperative delirium.

To determine the independent predictive factors for POD occurrence, binary logistic analysis was conducted. Items with *p* < 0.05 were included in the model analysis (because the ACCI score included whether there was diabetes, we used ACCI instead of “diabetes” and “using insulin/diabetic drugs”). The variables included in the model include age, MMSE score, ACCI score, preoperative CSF LCN2, and preoperative CSF IL-6. We found that lower preoperative MMSE scores were a risk factor for POD (OR 0.297, 95% CI 0.167–0.257; *p* < 0.001), whereas higher preoperative CSF LCN2 concentrations were an independent risk factor for POD (OR 2.546, 95% CI 1.345–4.822; *p* = 0.004) (Table 3). A forest plot displays the OR and 95% CI of the multivariate logistic regression analysis used to predict POD (Figure 4).

**Table 3:** Logistic regression analysis of factors of POD.

Variable	Regression coefficient	*p*-value	OR value	95% CI
Age	−0.007	0.841	0.994	0.932 ~ 1.059
MMSE	−1.216	<0.001	0.297	0.167 ~ 0.527
ACCI	0.305	0.155	1.357	0.891 ~ 2.065
Preoperative CSF LCN2	0.935	0.004	2.546	1.345 ~ 4.822
Preoperative CSF IL-6	0.013	0.529	1.013	0.973 ~ 1.055

OR, odds ratio; CI, confidence interval; MMSE, Mini-Mental State Examination; ACCI, Age-adjusted Charlson Comorbidity Index; LCN2, lipocalin-2; IL-6, interleukin-6.

### 3.5 Correlation of preoperative CSF LCN2 with CSF IL-6 and MDAS scores

To explore the potential mechanisms linking LCN2 to POD, we performed correlation analyses within the POD group (*n* = 29).

**Figure 5:**
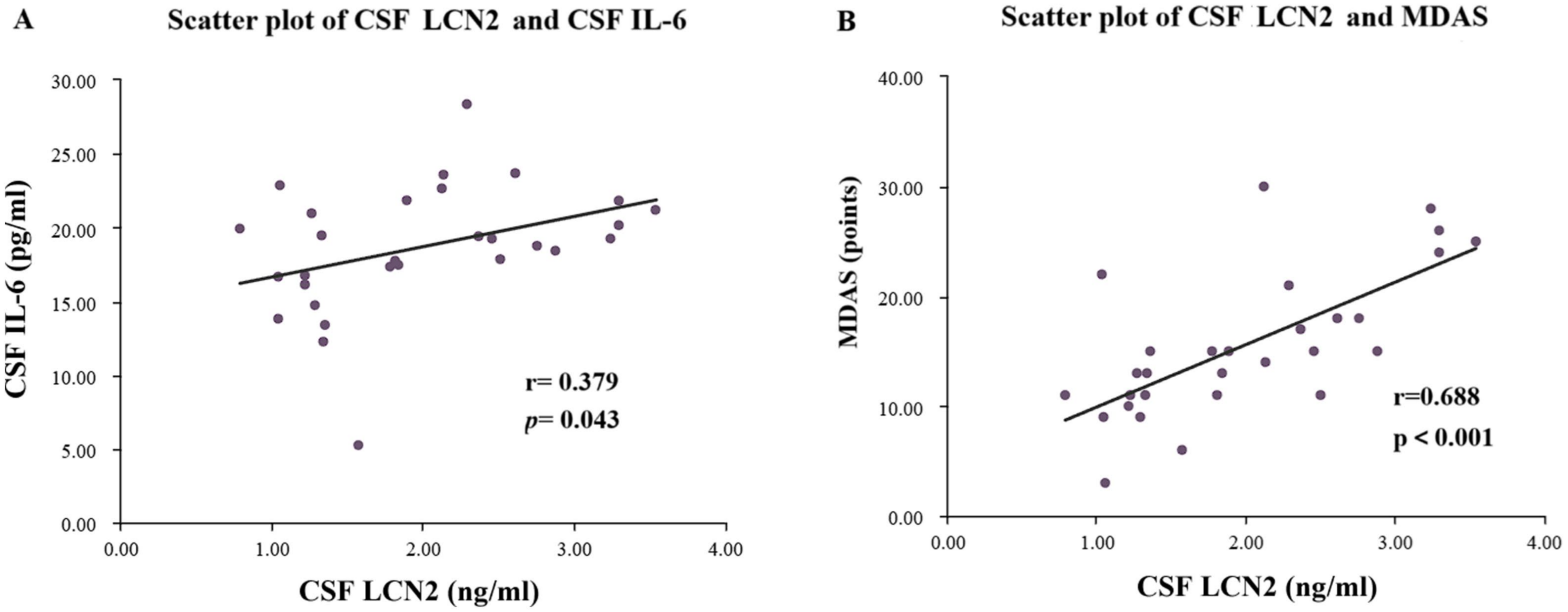
Scatter plots of LCN2 content and IL-6 content in CSF of the POD group patients, as well as scatter plots of LCN2 content in CSF and MDAS score. **(A)** Scatter plot of preoperative CSF LCN2 concentration and preoperative CSF IL-6 concentration. **(B)** Scatter plot of preoperative CSF LCN2 concentration and MDAS scores. POD, postoperative delirium; CSF, cerebrospinal fluid; LCN2, lipocalin-2; MDAS, Memorial Delirium Assessment Scale.

First, we investigated the relationship between CSF LCN2 and a key neuroinflammatory cytokine, IL-6. As shown in Figure 5A, a moderate but significant positive correlation was found between preoperative CSF LCN2 concentrations and preoperative CSF IL-6 concentrations (*r*_s_ = 0.379, *p* = 0.043) (Supplementary Table 1). This finding suggests that elevated LCN2 levels are associated with a heightened baseline neuroinflammatory state.

Next, we assessed whether CSF LCN2 levels correlated with MDAS scores. As depicted in Figure 5B, we observed a strong and highly significant positive correlation between preoperative CSF LCN2 levels and MDAS scores (*r*_s_ = 0.688, *p* < 0.001) (Supplementary Table 1). These correlations indicate that higher preoperative central LCN2 levels are not only predictive of POD occurrence but are also linked to a more severe clinical presentation of delirium.

### 3.6 Prediction performance of preoperative CSF LCN2 for POD

**Figure 6:**
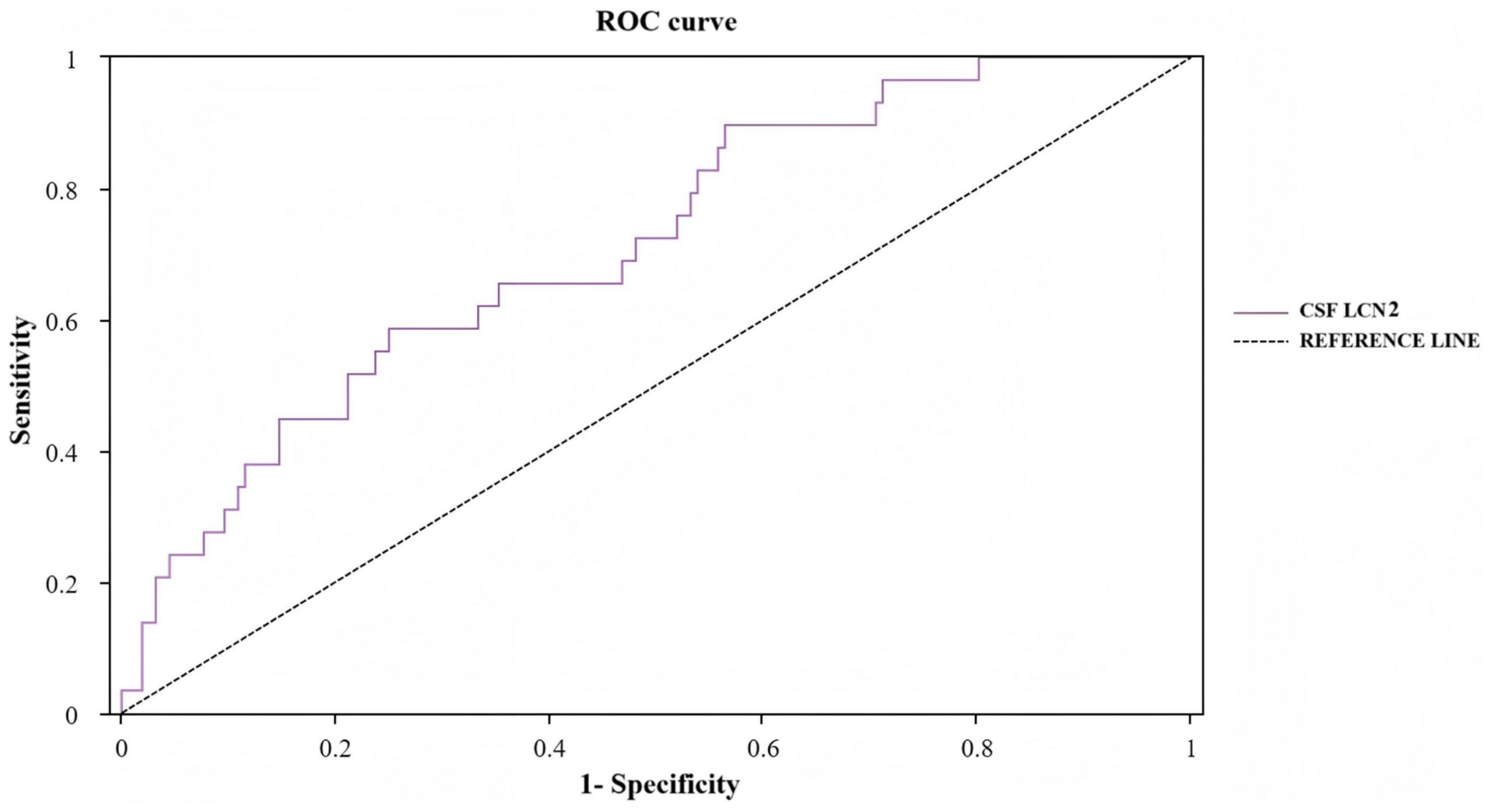
ROC curve for preoperative CSF LCN2 in POD. ROC, receiver operating characteristic; LCN2, lipocalin-2; POD, postoperative delirium.

We evaluated the predictive ability of preoperative CSF LCN2 for POD in older adults with hip fractures using ROC curve analysis. The results showed that preoperative CSF LCN2 had moderate predictive accuracy, with an AUC of 0.713 (95% CI 0.615–0.810; *p* < 0.001). According to the Youden index, the optimal cutoff value for CSF LCN2 was determined to be 1.769 ng/mL. At this threshold, the sensitivity and specificity of this biomarker for predicting POD were 58.6 and 75.0%, respectively (Figure 6; Table 4).

**Table 4:** The predicted values of preoperative CSF LCN2 for POD.

Variable	AUC	Sensitivity+ Specificity-1	Sensitivity	Specificity	Cut-off	Std. error	*p*	95% CI for AUC
Preoperative CSF LCN2	0.713	0.336	0.586	0.750	1.769	0.050	<0.001	0.615 ~ 0.810

AUC, area under the curve; CI, confidence interval; CSF, cerebrospinal fluid; LCN2, lipocalin-2; POD, postoperative delirium.

## 4 Discussion

Our investigation reveals that elevated preoperative CSF LCN-2 levels are associated with the development of postoperative delirium in older adults undergoing hip fracture surgery. Furthermore, within the POD cohort, preoperative CSF LCN2 concentrations correlated positively with both delirium severity, as measured by the MDAS score, and levels of the proinflammatory cytokine IL-6 in the CSF.

We found that 29 of 186 older adults with hip fractures developed POD, with a POD incidence of 15.6%, which is consistent with previous studies (46, 51, 52). The results of the present study showed that preoperative low MMSE scores, preoperative high IL-6 levels in CSF, and high ACCI scores were associated with the occurrence of POD. These results are also consistent with those of previous studies (10, 53–57). Neuroinflammation is considered one of the important mechanisms underlying the occurrence of POD (58). The significantly high levels of LCN2 and IL-6 in preoperative CSF strongly indicate that some individuals have a pre-existing, subclinical neuroinflammatory state upon admission. This state may reflect age-related cellular aging, early neurodegenerative processes, or the accumulation of chronic low-grade inflammation. Under multiple impacts such as fracture trauma and surgical anesthesia, peripheral inflammation cascades and amplifies to the CNS in the “fragile brain” population, leading to postoperative delirium.

It is also instructive to consider why some established risk factors, such as a history of stroke and intraoperative blood loss, were not significantly associated with POD in our multivariable analysis. This apparent lack of association may be attributed to several factors. For a history of stroke, its effect may be primarily mediated through variables already in our model, such as a lower baseline MMSE score or a higher ACCI score; once these powerful predictors were accounted for, the independent contribution of stroke history may have become attenuated. The nearly identical volumes of blood loss between groups (Table 1) likely reflect a highly standardized and effective surgical and anesthetic management, which minimized its variability and thus its potential as a risk differentiator in this cohort. Although we cannot exclude that a larger sample size might reveal a significant association (a Type II error), the lack of significance of these factors in our model serves to highlight the potent and independent predictive capacity of the preoperative neuroinflammatory state, as marked explicitly by CSF LCN2.

LCN2 has a role in various pathophysiological processes throughout the body, including inflammatory response and cognitive function (31, 39). Toll-like receptors activate downstream inflammatory cascades during the inflammatory response, which have been shown to upregulate LCN2 (17). The LCN2 promoter has a common site for NF-κB, a transcription factor activated by various inflammatory cytokines (59). LCN2 seems to be associated with many neurodegenerative diseases, and there is evidence to suggest that LCN2 can participate in the pathophysiology of neurodegenerative diseases by affecting pathways such as inflammation, cell death/survival signaling, and iron metabolism (60). A meta-analysis showed that the peripheral blood LCN2 concentration in AD patients was significantly elevated compared with that in the control group. Peripheral blood LCN2 levels are also elevated in patients with mild cognitive impairment (35). Changes in LCN2 are associated with decreased executive ability in AD patients (61). A positive correlation between LCN2 and amyloid *β*-42 in CSF has been recognized, especially in MCI patients. LCN2 in CSF may serve as a predictive biomarker for the transition from MCI to AD dementia (38). Studies have shown that elevated plasma LCN2 levels are associated with non-motor symptoms in patients with Parkinson disease, and their mediated neuroinflammation is associated with cognitive impairment in patients with Parkinson disease (62).

The role of LCN2 in postoperative cognitive impairment remains to be elucidated. In rat models of POD, peripheral and central LCN2 concentrations increase after cardiac and abdominal surgery (41). Correlation analysis shows that spatial learning ability is correlated with plasma and hippocampal LCN2 levels (40). A prospective cohort study showed that the difference between postoperative and preoperative plasma LCN2 levels was associated with the occurrence of POD, with a greater difference observed in the POD group (42). We found that in both POD group and non-POD group patients, postoperative CSF LCN2 levels were significantly higher than preoperative levels. However, contrary to previous findings, we found no significant difference in the postoperative-to-preoperative change in LCN2 levels between patients with and without POD. This discrepancy might be attributable to differences in the patient populations; our cohort was comprised exclusively of older adults with hip fractures, who endure greater preoperative pain and stress. Differing anesthetic techniques and surgical durations may also have contributed to the discrepancy.

Strengths of the present study include its prospective design, which minimizes recall and selection bias. By focusing on a specific, homogenous population of older adults with hip fracture, we minimized baseline variability. Crucially, the exclusive use of spinal anesthesia for all participants is a major strength because it effectively eliminated the significant confounding influence that different anesthetic techniques (i.e., general vs. regional anesthesia) can have on POD outcomes. This rigorous standardization enhances the internal validity of the observed association between CSF LCN2 and POD.

Several limitations should be acknowledged. First, because this was a single-center study, the generalizability of our findings may be limited. Second, the relatively small sample size may have limited the statistical power to detect smaller effects. Therefore, multicenter, large-sample prospective studies are warranted to validate our findings. Third, although we controlled for key confounders, the observational nature of the study precludes definitive conclusions about causality, and residual confounding may exist. Fourth, the invasive nature of CSF collection limits the direct clinical translatability of CSF LCN2 as a routine screening tool. Finally, our panel of inflammatory markers was limited, and we did not measure plasma cytokines. Future research to address these limitations is warranted, including relevant basic research to further verify and expand on the findings. Animal models of hip fracture could be employed to mechanistically dissect how LCN2 contributes to neuroinflammation and delirium-like behaviors, providing a crucial bridge from clinical observation to molecular pathophysiology.

## 5 Conclusion

Elevated preoperative CSF LCN2 concentrations are associated with an increased risk of POD in older adults undergoing hip fracture surgery. Moreover, in patients with POD, the preoperative CSF LCN2 level is positively correlated with preoperative CSF IL-6 concentration and with MDAS scores.

## Data Availability

The original contributions presented in the study are included in the article/Supplementary material, further inquiries can be directed to the corresponding authors.
