# Supplementary material for: Lipocalin-2 in preoperative cerebrospinal fluid is a biomarker for postoperative delirium after hip fracture surgery in older adults: a prospective cohort study
Source: Front Neurol. 2025 Sep 12;16:1653407. doi: 10.3389/fneur.2025.1653407 (PMC12465281; doi:10.3389/fneur.2025.1653407)
Supplement: Supplementary file 1 [file Table_1.DOCX]

|  | | | |
| --- | --- | --- | --- |
|  |  | MDAS | Preoperative CSF IL-6 |
| Preoperative CSF LCN2 | Correlation coefficient | 0.688** | 0.379* |
|  | *p* value | 0.000 | 0.043 |
|  | Sample size | 29 | 29 |

**Supplementary table 1.** Correlation analysis of cerebrospinal fluid LCN2 and cerebrospinal fluid IL-6, as well as MDAS score in POD group patients.

LCN2, lipocalin-2; IL-6, Interleukin-6; MDAS, Memorial Delirium Assessment Scale; POD, postoperative delirium.
